# Ambidextrous Relationships and Social Capability as Employee Well-Being: The Secret Sauce for Research and Development and Sustainable Innovation Performance

**DOI:** 10.3390/ijerph17093072

**Published:** 2020-04-28

**Authors:** Lucía Muñoz-Pascual, Jesús Galende

**Affiliations:** Multidisciplinary Institute for Enterprise (IME), Department of Business Administration and Management, University of Salamanca, Campus “Miguel de Unamuno”, Building FES, 37007 Salamanca, Spain; jgalende@usal.es

**Keywords:** sustainable innovation performance, research and development, creativity, informal/formal relationships, ambidextrous relationships, social capability

## Abstract

This study examines the effects that ambidextrous relationships, i.e., oriented relationships within a firm towards the development of exploitation activities and oriented relationships towards the development of exploration activities, have on employee performance in terms of creativity, research and development (R&D), and sustainable innovation performance. We contend that informal relationships affect employee creativity and R&D. However, formal relationships affect employee creativity and sustainable innovation performance. The purpose of this study is to inject new positive relationships into firms by examining ambidextrous relationships and the moderating role of social capability in the relationships–innovation effect. We empirically tested our hypotheses by using multisource data collected from 245 Spanish firms across 14 industries. Our structural equation models suggest that these two types of relationship predict employee creativity, which in turn increases sustainable innovation performance and R&D. The results reveal that the effects between informal relationships and creativity are stronger, as are the effects between formal relationships and sustainable innovation performance. A multigroup structural analysis also reveals that effects between informal and formal relationships, employee creativity, R&D, and sustainable innovation performance are stronger within firms that have employees with high social capability. The efforts and investments made in employee social capital support the development of new ideas, R&D, and innovation success. Support is provided for an ambidextrous Human Resource Management (HRM) approach which is relevant for innovation, and several practical recommendations and implementation guidelines for Small and Medium Enterprises (SMEs) are provided. This study provides a plausible explanation of two important management mechanisms for enhancing creativity—R&D and sustainable innovation performance. Relationships are malleable, and this study suggests that fostering formal and informal relationships might hold the key to sustainable innovation performance and unlocking desirable employee behavior.

## 1. Introduction

Firms from industrialized countries are experiencing constant changes in the competitive context that leads to the strengthening of certain factors, mainly those focused on the innovation. The main action is to invest in the construction of technological capabilities and relationships offered for the generation of ideas, knowledge, and skills necessary to develop new products [1,2,3,4,5,6,7].

Following the study by Gratton and Ghoshal [8] concerning human capital dimensions as antecedents of innovation (intellectual capital, emotional capital, and social capital), this study examines the impact of social capital on creativity, on sustainable innovation performance, and on research and development (R&D) projects. 

Indeed, the development and diffusion of sustainable innovations by companies has been deemed necessary for the successful application of new relationships, thus favoring social capability while boosting economic growth [9]. 

Accordingly, sustainable innovations represent a means through which organizations can foster new relationships between different parts [10]. 

The previous arguments emphasize that more research is needed to fully comprehend the interrelated nature of firms’ innovation dynamics and relationships. 

Furthermore, R&D and innovation requires a “society pool” approach whereby different stakeholders (e.g., employees, customers, suppliers, and governments) are involved [11,12,13]. On the one hand, stakeholder involvement helps to clarify the criteria for outcomes and then operationalizes new strategic practices for innovation according to these criteria. A clear example of relationships, creativity, and innovation is currently developing in small, medium, and large companies around the world. Faced with the current health and economic crisis suffered by Covid-19, employees are using their highest levels of relationships, teamwork, and social capacity to adapt and develop new sanitary materials and instruments in record time. If we go one step further and get social relationships to cross borders, we can also achieve broader R&D and innovation results, such as the development of a new treatment against Covid-19 or the creation of a new vaccine [14,15].

The study, supported by Social Systems Theory [16], considers two types of internal social networks as antecedents of employee creativity and their effects on innovation performance (IP) and R&D. We propose two ambidextrous social networks as antecedents of employee creativity: (a) “informal relationships”; and (b) “formal relationships” at individual level. Creativity is examined as an antecedent capability of IP and R&D. IP and R&D are examined at organizational level. As a relevant contribution in the measures of the variables, we propose two measures for IP (product innovation and process innovation) and another measure for R&D. The measures taken ensure robustness and confidence in the model and its results, resolving inconsistency of results between IP and R&D [17]. Finally, we propose social capability (SC) as a moderating variable at individual level, supported by the Dynamic Capabilities View [18].

Following Alegre and Chiva [19], the study applies a complex quantitative method for hypotheses testing: structural equation modeling (SEM) and multigroup structural analysis. SEM is a complex statistical technique for studying relationships between variables with direct and indirect effects. Our study therefore provides a unique, complete, and coherent understanding of R&D and IP. The study adopts an online survey, gathering data from 245 Spanish technology firms in 14 different industries. The surveys were managed online using the SurveyMonkey (payment platform). To minimize the probability of errors due to the interpretation of the language used, the survey was initially drafted in English, and the back-translation method was used for the survey items. Therefore, the questionnaire was originally written in English, translated into Spanish by a certified translator, and then back-translated into English. Before the survey, a pre-test was carried out with five prestigious scholars and managers who helped to draft the final survey. Finally, the firms were contacted by telephone to introduce the study, and mass mailings of the survey were then sent to them. The survey took 20 min, and the respondents were the CEOs within each firm.

Most investigations have been developed usually in one sector that we can qualify as of medium or high technological profile, i.e., where a greater intensity in knowledge predominates (medium or high tech). In this study, we have obtained a population of highly innovative firms from 15 very different sectors (agriculture and livestock, manufacturing, power and gas supply, water supply and pollution, building, vehicle trade and repair, transport and storage, catering, information and communication, housing, scientific activities, administrative activities, health activities, and other services) [20]. These firms have received funding from the Spanish government for the development of R&D projects in the last five years.

The findings reveal important advances for managers, including alternative social networks that lead to IP or R&D [21]. First, informal relationships and formal relationships impact positively on employee creativity. Secondly, creativity impacts positively on IP but there is no evidence regarding R&D projects. Thirdly, there is no evidence for informal relationships leading to IP but there is evidence for informal relationships leading to R&D. Fourthly, formal relationships lead to IP but there is no evidence for formal relationships leading to R&D. Finally, the study reports the findings on the moderating role of SC level in the model. Usually, SC significantly improves the explanatory power of the model. Therefore, employees with higher SC are related to the implementation of: (a) informal relationships and creativity, facilitating R&D; and (b) formal relationships facilitating IP.

In summary, the main contribution that this study makes to the literature is the study of the ambidextrous relationships of employees, i.e., oriented relationships within the firm towards the development of exploitation activities and oriented relationships towards the development of exploration activities for innovative success. Ambidextrous relationships are antecedents of IP and R&D. We propose an original structural equation model and adopting scales previously validated in other fields, such as the Torrance Test of Creative Thinking (TTCT) [22,23], including variables at individual and organizational level. Therefore, this study makes an original contribution when proposing the model between both types of relationships, creativity, and sustainable innovation. This study helps the business world by giving it the opportunity to observe when and how it has to promote ambidextrous relationships at the same time, and formal relationships or informal relationships according to the part of the innovative process that is being developed (creation of ideas, project development, or product innovation). These employee relationships, which are essential for the adaptation and survival of the firm, can be derived in the search for new opportunities (exploration activities) and in the search for more immediate advantages (exploitation activities). Therefore, the development of ambidextrous relationships (formal and informal) allows increasing creativity by fostering the ability to explore and increase the employee capabilities in a particular area and boost exploitation skills in that area [24].

Therefore, the aim of this study is to answer three original research questions: Can employees develop new ideas with management of ambidextrous relationships? Can firms develop sustainable IP and R&D with management of ambidextrous relationships? Can SC of employees influence the development of IP and R&D?

## 2. Theoretical Framework 

The main theories of this research are Social Systems Theory [16] and the Ambidextrous Organizational Approach [25].

### 2.1. Social Systems Theory

According to Luhmann [16], the basic element of all social systems is communication between individuals, or the synthesis of utterances (including physical movements as well as speech or writing), information, and understanding, i.e., the relationships. A relationship is a social operation, which cannot be reduced to individual action [16]. A relationship requires an interaction between at least two individuals, and from a sociological perspective it is the understanding or feeling rather than the utterance or intention that determines the significance or outcome of the relationship (formal or informal and weak or strong). 

This conceptualization of social systems, as composed of relationships and communications, has implications for the management of change and innovation. Since relationships cannot be treated as the products of individual actors but as the processes, creativity, and innovation must be explained on the basis of the logic of the relationships system. It is the relationships system management that determines, through its processes, what reasons or types of relationships come about [21,26].

### 2.2. Ambidextrous Organizational Approach

Gibson and Birkinshaw [25] show that managers and employees can direct resources towards exploration and/or exploitation activities. Jarzabkowski et al. [27] argue that firms should create the context for employee ambition to flourish. Although the ambidextrous literature recognizes the important role employees play in guiding their resources towards exploration and/or exploitation activities, most have focused their studies on the organizational context or climate as generators of ambidextrous behaviors. It is, therefore, important to study the Ambidextrous Organizational Approach on an individual level [28].

Rogan and Mors [29] have highlighted the important role played by Human Resource Management (HRM) within the Ambidextrous Organizational Approach. Employees must be managed to obtain ambidextrous behavior in the firm. Brusoni and Rosenkranz [30] argue that it is essential in changing environments for employees to have a high level of ambidextrous behavior. Ambidextrous HRM should be a priority for managing managers [31]. Managers must be responsible for coordinating HRM towards exploration and/or exploitation activities. Jansen et al. [32] and Alghamdi [33] have reported that ambidextrous HRM helps to generate greater group cohesion and a more proactive attitude towards innovation.

## 3. Conceptual Framework and Hypotheses

### 3.1. Sustainable Innovation Performance and Research and Development

Innovation is the generation, assimilation, and exploitation of a novelty or technical change. The Oslo Manual [34] considers innovation as the introduction of a new or significantly improved technical change in products (services). Other authors, such as Wang and Ahmed [35], argue that innovation is the introduction of technical change or new solutions to problems. We have studied sustainable IP, i.e., product IP and process IP for a long time.

However, R&D is based on creative work carried out systematically to increase knowledge and the use of that knowledge to create new applications. It is composed of three activities: basic research, applied research and experimental development. R&D is based on both formal R&D and informal or occasional R&D carried out in other departments [34].

### 3.2. Employee Relationships and Ambidextrous Relationships

Employee relationships are the networks and contacts established by the members of the firm among themselves and with other external agents. They can be of two types: weak ties (formal) and strong ties (informal). Weak ties occur between the members of the organization or with other external agents as a result of the formal and contractual relationships established within the work environment. Strong ties occur between the members of the organization or with other external agents as a result of informal relationships, relaxed environments that go beyond mere labor relationships and that serve to acquire trust, commitment, and attachment to the organization or well-being [36].

Therefore, ambidextrous relationships are oriented relationships within the firm towards the development of exploitation activities and oriented relationships towards the development of exploration activities for creativity, R&D, and sustainable IP. As mentioned before, these employee relationships, which are essential for the adaptation and survival of the firm, can be derived in the search for new opportunities (exploration activities) and in the search for more immediate advantages (exploitation activities). Therefore, the development of ambidextrous relationships (formal and informal) allows increasing creativity by fostering the ability to explore and increase the employee capabilities in a particular area and boost exploitation skills in that area [24].

### 3.3. Creativity and Social Capability: Keys to Innovation Success

Relationship effects on innovation and R&D are not independent of contextual variables or employee capabilities, among others. In addition, within contextual variables there are different levels of analysis (individual, group, and organizational). All are necessary for the analyses of innovation and R&D [37]. This study examines creativity and SC. Both are considered factors that intervene between employee relationships and IP and R&D.

Creativity can be defined as the production of new ideas about practices, products, or processes that will be useful to firms for developing new products or processes [38]. Individual creativity is analyzed here [22]. The creativity of employees is considered to be a predecessor of technological innovation. Runco and Jaeger [39] argue that creativity requires originality and effectiveness. Originality is vital, but must be balanced appropriately. Effectiveness may take the form of value. This label is quite clear in the economic research on creativity; it describes how original and valuable products and ideas depend on the current market, and more specifically on the costs and benefits. Creativity is an important channel between relationships and IP and R&D [40]. Work teams may be able to generate environments and routines in which creativity is developed, whereby it may be considered a dynamic capacity developed to obtain IP and R&D [41]. Creativity is an intervention mechanism between relationships, R&D, and IP, and it is an important channel. It can be considered a dynamic capacity that the employees and, therefore, the company will have, prior to obtaining innovation results.

Some authors [22] have identified four characteristics of a creative person in an organization: fluency, flexibility, elaboration, and originality. Zollo and Winter [42] argue that skills will help to generate mechanisms or ideas for greater effectiveness in firms. Accordingly, and before improving their IP, firms will have highly creative individuals, with patterns and capabilities. Consequently, and depending on the complexity, competitive environment or dynamism, employees may generate more or less ideas [43]. Creativity is clearly affected by relationships.

Additionally, if competitive advantage is based on the accumulation of resources and strategic capabilities, creativity may be considered a source of competitive advantage [44]. Therefore, considering creativity as an aspect to train and develop within the company, it is possible to argue that companies that implement an HRM system based on relationships help to develop creativity. 

On the other hand, SC can be defined as the ability to have or acquire certain capabilities destined to overcome economic and social changes. Those employees, who have a certain level of socialization capacity, will have a participatory attitude in the company, will promote union, and will influence both their own and co-worker attitudes. Therefore, SC can be an instrument to generate more ideas and, consequently, improve IP and R&D [36].

Thus, the ability to socialize is a personal competence that the worker has or can develop to a greater or lesser degree, and the development of this competence will make the investment that the entrepreneur makes in certain practices such as the enhancement of social relationships between employees and members of the organization, to a greater or lesser extent [36,45,46].

In addition, employees with a high level of SC will be a complementary asset for the firm’s development of IP and R&D. SC is therefore an important capability for obtaining a competitive advantage, given its imperfect imitability [19].

### 3.4. Positive Effects of Ambidextrous Relationships on Employee Creativity, Sustainable Innovation Performance, and Research and Development

This study goes a step further by identifying, measuring, and testing the type of employee relationships that enhance employee creativity and, consequently, IP and R&D. First, informal relationships refer to all the contacts that employees make between themselves and with other agents. e.g., meetings outside the workplace, group sessions. These relationships are not included in a formal document such as a labor contract. Second, formal relationships are easier to manage than informal relationships because they are based on the work environment, where the main reason for the relationship is the exchange of labor information.

The accumulation and management of both types of relationships can be important for the development of creativity and/or innovation and R&D [45,47,48].

Most firms are concerned with managing employee relationships that fall within professional competencies, i.e., formal relationships [49]. These can be important sources of innovation generation within the firm, but it is necessary to emphasize that employees can take labor relationships far beyond the formal limits of the firm. This can lead to more lasting informal relationships among employees that help to strengthen links, ties, and complementary knowledge relevant to the phases of the innovative process [50]. In this line, authors such as Phene et al. [51] find that employee informal relationships outside the workplace are vital for the development of new ideas, products, and processes. Informal contact networks can generate a greater number of ideas and knowledge and, therefore, greater possibilities for innovation. A relaxed work environment, where a good work environment prevails and trust between the members of the organization exists, is the ideal context for the development of new ideas [52].

However, both types of relationships (informal and formal) can be important for cooperation processes, and essential in the development of group ideas, innovation results, and R&D. In addition, authors such as Adams et al. [49] also incorporate as formal relationships those that occur between these and other agents that are related to the firms (suppliers, customers, and allies). These authors argue that these are marked relationships within their professional competences, i.e., formal relationships that can generate important sources of knowledge, ideas, R&D projects, and numerous innovation results.

Therefore, the following hypotheses are proposed:

**Hypothesis** **1.** *Informal relationships are positively related to employee creativity.*

**Hypothesis** **2.** *Formal relationships are positively related to employee creativity.*

However, Amabile et al. [53] state that the creativity of employees can generate any type of technological innovation (product and process) and R&D. Van de Ven [54] indicates that creativity is one of the facilitating factors of the innovative process. For this reason, the following research hypotheses are proposed:

**Hypothesis** **3a.** *Employee creativity is positively related to sustainable innovation performance.*

**Hypothesis** **3b.** *Employee creativity is positively related to R&D.*

Regarding the possible direct effects that can occur between both types of relationships and IP and R&D, some authors have studied the contractual relationships between employees and other external agents [55], but they did not analyze informal relationships. Therefore, our study provides great value when proposing hypotheses concerning informal relationships, creativity, IP and R&D.

Authors such as Gratton and Ghoshal [8], Byrne et al. [56], and Eva et al. [57] argue that close informal networks among employees are direct sources of innovation and R&D. In this way, through mutual trust, engagement, internal values, cooperation, or the exchange of knowledge, important results can be obtained in terms of innovation and R&D [58]. Specifically, authors such as Gratton and Ghoshal [8] argue that aspects such as relaxed meetings, consensus, and cooperation are factors that positively influence product innovation. Others, such as Birkinshaw et al. [59] argue that informal, deep, and long-term relationships among employees are powerful resources for the development of innovation. 

On the other hand, authors such as Tödling et al. [50] reveal that formal relationships help the development of product and process innovation. From formal relationships, new technical and applied knowledge can be obtained that can be embodied in new innovation processes and products. Matarazzo and Finkelstein [60] argue that the formal exchange of ideas and knowledge among employees, customers, competitors, or suppliers can contribute positively to the innovation process. It is considered relevant to introduce a new effect between both types of relationships and R&D [55]. Considering that both types of relationships can also influence previous phases of the development of new innovations, such as the search for information, project, experimentation, i.e., to R&D, it will be possible to know in which phases of the innovative process it is more relevant to invest in according to the types of relationships. It must be taken into account that informal relationships may be relevant during the initial stages of R&D and formal relationships during more advanced phases of R&D and innovation. 

Therefore, we propose:

**Hypothesis** **4a.** *Informal relationships are positively related to sustainable innovation performance.*

**Hypothesis** **4b.** *Informal relationships are positively related to R&D.*

**Hypothesis** **5a.** *Formal relationships are positively related to sustainable innovation performance.*

**Hypothesis** **5b.** *Formal relationships are positively related to R&D.*

Therefore, the Ambidextrous Organizational Approach considers relationships with two orientations (exploration and exploitation). Relationships arise when an individual is connecting with another person. Consequently, relationships arise not only from formal relationships with partners or a boss, but also from informal relationships with partners and friends within firms. There are several types of relationships: informal and formal, strong and weak, etc. This study focuses on informal and formal relationships. Some authors, such as Hayton [61] and Paton [62], report a positive effect between relationships and the generation of new ideas and innovation. For example, diversity and relationships might make people search for new knowledge and new cognitive approaches that promote creativity and innovation. 

Dyer and Shafer [63] show that individuals with contacts generate major creative and innovative ideas. Similarly, Sung and Choi [64] support the idea that team relationships increase creativity. Therefore, relationships and communication are key indicators. Moreover, this study goes a step further by analyzing ambidextrous relationships: informal and formal. Although it is true that informal relationships are very important for the development of creativity and innovation, we should not forget formal relationships, as we highlight throughout the study. 

We posit that relationships should not be only regarded from a formal perspective, as we are also interested in the potential impact of an informal perspective on creativity and innovation [65]. The knowledge obtained from informal meetings can generate new capabilities and it can be oriented towards the exploration activities, and so increase the probability of being creative and innovate [23,66]. Formal relationships are contractual relationships with an orientation towards the exploitation activities, e.g., meeting with boss, meeting in conferences, etc. However, employees need technical meetings to create new ideas in their fields. Therefore, authors such as Poon et al. [67] and Wipulanusat et al. [68] show that ambidextrous behaviors, culture, and relationships positively influence creativity and innovation.

### 3.5. Social Capability as a Moderator

This study goes a step further by introducing employee SC. SC can further develop the generation of new ideas, fostering the creation of new projects or innovations. If an employee has a high social capacity, they will be alert, capturing knowledge and integrating it into new ideas or new innovation processes [19,69,70].

Several arguments justify the use of SC as a moderating variable between relationships, creativity, R&D, and IP. 

First, employees not only accumulate knowledge within the organization (stock), but they also possess, develop, and hone skills such as their SC (flows), which help strengthen relationships for the development of a new idea, a new project, or a new product.

Second, we can justify the existence of SC as a moderating variable if we consider it a complementary asset. Those employees that achieve a certain degree of SC will help the organization obtain a competitive advantage. From this perspective, firms also need employees that have developed their SC [71].

Van de Ven [54] argued that among the facilitators of the innovative process are the context, culture, or human capabilities that help to achieve innovative performance. Others such as Muñoz-Pascual et al. [70] show that environmental practices can help to product innovation success. Pérez [36] and Yun and Lee [72] argued that the ease with which employees are able to perform favors, integrate into work teams, or converse voluntarily and with their co-workers can enhance existing relationships between members of the firm and, consequently, innovation and R&D. This group cohesion and closeness helps to achieve the goals of the firm. Tsai and Ghoshal [73] added that the communication capability, attendance at social events and meetings, or knowledge of social norms can help to establish strong relationships and therefore, innovative success and R&D is achieved. Aiman-Smith et al. [74] and Hegde and Shapira [75] indicate that firms supporting learning capability and SC will achieve innovation success more easily. SC may have a positive influence on innovation. Finally, Subramaniam and Youndt [76] argue that learning capability and SC helps strengthen relationships, R&D, and PIP. 

Therefore, a contingent relationship shows the effects that SC can have on the research model [19,77]. Our model suggests that employees with a high SC facilitate innovation results.

Therefore, we propose:

**Hypothesis** **6.** *Social capability moderates the relationships between informal/formal relationships and creativity, sustainable innovation performance, and R&D so that relationships are stronger in firms with a higher level of social capability.*

## 4. Methods

### 4.1. Study Design: Sample and Data Collection

We applied a quantitative method to test for IP and R&D (Hypotheses H1–H6). Research as a way of knowing, interpreting, and transforming reality cannot ignore the constant demands of an increasingly unstable, complex, and diverse business world; hence, the traditional research approaches fall short in the resolution of problems and situations that require new perspectives. In this context, the application of SEM is important, and it allows us to exploit the strengths of model to achieve more complete understandings of relationships between employees within organization. 

SEM is a complex statistical technique for studying relationships between variables with direct and indirect effects. In addition, we used a multigroup analysis for testing one moderating variable in the model (SC). Our study therefore provides a unique, complete, and coherent understanding of R&D and PIP. Analyzes using SEM allow a series of dependency relationships to be examined simultaneously, and are particularly useful in our models when a dependent variable (employee creativity) becomes an independent variable in subsequent relationships of dependency. SEM models have high capacities compared to other multi-variant techniques: (1) These models can estimate and evaluate the relationship between unobservable constructs (latent variables). A latent variable is an unobservable construct that is assumed by the researcher (creativity, social capacity, etc.), which can only be measured using observable variables [22] in the case of creativity SEM models allow the use of multiple measures to represent the construct and control the specific measurement error of each variable, i.e., the researcher can evaluate the validity of each measured construct (2) Another important characteristic of SEM is that it provides a set of indices that determine whether the proposed theoretical structure provides a good fit to the empirical data. Therefore, the use of SEM has allowed us to evaluate and test our theoretical models and has helped us to contrast the hypotheses initially proposed. We follow the phases and basic assumptions to apply SEM: (a) specification, (b) identification, (c) estimation of parameters, (d) evaluation of the fit (*p*-value and RMSEA (root mean square error of approximation), (e) re-specification of the model and f) interpretation of results. To apply SEM, we have used the Analysis of Moment Structures (AMOS) program. This tool was created by Arbuckle [78] and has allowed us to specify, view and modify structure models graphically in a simple way.

A sample was drawn from innovative Spanish private business firms across 14 different industries listed in the database of the Centro para el Desarrollo Tecnológico e Industrial (CDTI) and Sistemas de Análisis de Balances Ibéricos (SABI) for 2016, 2017, and 2018. Therefore, it is a cross-sectional study for three years. These innovative companies were selected because they have received funding for R&D projects from the Spanish Ministry of Economy and Competitiveness for three consecutive years. The information was collected through an ad hoc online survey (SurveyMonkey payment platform). The initial population consisted of 1446 firms across 14 different industries. This screening procedure yielded a final sample of 245 firms that participated in the data gathering and provided sufficient information for the analysis. These data represent a 16.94% response rate, with a sampling error of ±5.71% at a confidence level of 95%. If we compare the response rate with previous studies on creativity and innovation we see that 16.94% is a very acceptable response rate for studies within this field [1,66].

Before the survey, a pre-test was carried out with five prestigious scholars and managers who helped to draft the final survey. The survey took 20 min, with the respondents being CEOs within each firm. The CEOs are responsible for decision-making and they know all the information about the employee relationships and IP. In this sense, the CEOs have an objective and realistic view of the employee tastes and preferences to manage the relationships and, in addition, they know the situation of the firm for IP and R&D. As in any innovative process, we need to know information about all the employees involved in the innovation phases. The CEO gives us a global and complete vision of the complex network of relationships that all employees involved in this process have from the generation of ideas to the final development of the product. It is relevant to know the relationships of employees among all professional categories within the firm.

Our final sample of 245 firms represents 14 industry categories: agriculture and livestock (N = 4), manufacturing (N = 110), power and gas supply (N = 3), water supply and pollution (N = 3), building (N = 10), vehicle trade and repair (N = 27), transport and storage (N = 2), catering (N = 2), information and communication (N = 27), housing (N = 1), scientific activities (N = 47), administrative activities (N = 5), health activities (N = 3), and other services (N = 1). Most investigations have been developed usually in one sector that we can qualify as of medium or high technological profile, i.e., where a greater intensity in knowledge predominates (medium or high tech). We have obtained a population of highly innovative firms from 14 different sectors [20]. Our model can be tested in different industrial sectors, which generates greater variability and richness in the obtained results. We do not limit ourselves to a single innovative sector, but to the study of innovative firms within several sectors. To try to control the possible differences between the firms located in the different sectors, we take as condition that the population of firms belonging to this study are those that in the last five years have received funding from the Spanish government for the development of R&D projects [1,66].

Table 1 provides a summary of the demographic information.

### 4.2. Measures

Data were collected from multiple sources and analysis levels: (a) individual level: employee relationships, employee creativity, and SC measured through the perceptions and management tools of the CEO; and (b) organizational level: IP and R&D. All the variables were rated on a seven-point Likert-type scale, ranging from 1 (strongly disagree) to 7 (strongly agree).

#### 4.2.1. Employee Relationships (REL)

The items of relationships (REL) were measured with 15 items grouped into two dimensions: eight items for informal relationships (INFOR_REL) and seven for formal relationships (FOR_REL), considering the weak and strong ties that encourage employees to work towards their goals [55,79].

#### 4.2.2. Employee Creativity (EMPL_CREA)

Employee creativity (EMPL_CREA) is a one-dimensional variable with seven items: four items adapted from the TTCT [22] and three items of our own according to the perceptions, knowledge, and management tools of the CEO. The TTCT is perhaps the most widely used instrument within the international context of education and psychology for the identification of creative abilities. The TTCT evaluates creative thinking at an individual level through different tests of verbal and figurative production. These tests are evaluated according to fluency, originality, elaboration, and flexibility; thus, contributing a new practical application of TTCT in the field of management.

#### 4.2.3. Social Capability (SC)

SC is a one-dimensional variable with ten items was used according to perceptions, knowledge, and management tools of the CEO (social norms, networking etc.) [36].

#### 4.2.4. Sustainable Innovation Performance (IP)

IP was measured by six items grouped into two dimensions: three items for product IP [35,79] and three items for process IP [19,80].

#### 4.2.5. Research and Development (R&D)

R&D is a one-dimensional variable with three items: number of employees dedicated to R&D, R&D expenses and hours dedicated to R&D [67].

#### 4.2.6. Control Variables

Two control variables are considered: size and age [81]. Firm size and age are critical firm-specific factors that can affect IP and R&D. 

Table 2 provides a summary of the model’s main scales.

## 5. Data Analysis and Results

### 5.1. Descriptive Statistics

Pearson correlations indicate the degree of relationships between two variables, both quantitative. This coefficient is a measure that indicates the relative situation of the events with respect to the two variables, i.e., it represents the numerical expression that indicates the degree of correspondence or relationship that exists between the two variables. These numbers vary between limits of +1 and −1 [82]. For the use of descriptive analysis, we have used the SPSS 23 program. 

Pearson correlations, means, and standard deviations for the study variables are in Table 3.

### 5.2. Analysis of Variance Test

To check for the presence of non-response bias, an Analysis of Variance test (ANOVA) with SPSS 23 program of differences of means was carried out in which relevant aspects that could affect the sample’s behavior were compared subsequent to the statistical analyses, such as size (headcount) and seniority (age), among 50 late respondents (n = 50 late respondents) and 50 early respondents (n = 50 first respondents) [83]. The ANOVA did not reveal any significant differences between early and late responses, rejecting the null hypotheses with no differences between the mean headcount (F = 1.672, *p* = 0.199) and seniority (age) (F = 0.041, *p* = 0.840). It may be concluded that there is no non-response bias, and the sample suitably represents the population.

In addition, a new analysis of variance was performed where we compared whether size, age and business organization from respondent (n = 245) and several non-respondent firms (n = 245) can have significant differences. In this second analysis, the results reveal again that there are not significant differences in age (F = 0.149, *p* = 0.700), size (F = 0.435, *p* = 0.511) and business organization (F = 0.036, *p* = 0.850). Therefore, the null hypotheses are rejected, and the sample is representative of the population.

### 5.3. Factor Analysis

The factor analysis provides a good fit [82]. For the use of factor analysis with axis rotation we have used the SPSS 23 program.

As Table 2 shows, REL consists of two factors, with 62.22% of explained variance and a Cronbach’s alpha of 0.91 (INFOR_REL) and 0.88 (FOR_REL); EMPL_CREA consists of one factor, with 75.09% of explained variance and a Cronbach’s alpha of 0.94; IP with 73.69% of explained variance and a Cronbach’s alpha of 0.68 (product IP) and with 78.26% of explained variance and a Cronbach’s alpha of 0.85 (process IP); R&D consists of one factor, with 90.85% of explained variance and a Cronbach’s alpha of 0.95; finally, SC consists of one factor, with 68.23% of explained variance and a Cronbach’s alpha of 0.90. For the internal consistency analysis, we have used Cronbach’s alpha with the SPSS 23 program as Table 2 shows. The index shows a good internal consistency. 

Additionally, the Confirmatory Factor Analysis (CFA) (first and second order) was needed to test the one-dimensionality of the measures within variables with different dimensions (REL). For relationships, the two-factor model recorded a good fit with the data [χ2/df = 2.70; the Comparative Fit Index (CFI) = 0.95; the Incremental Fit Index (IFI) = 0.95; the Root Mean Squared Error of Approximation (RMSEA = 0.08] (*p* < 0.001). 

It may be concluded that the items used to measure REL, CREA, IP, and SC are suitable. They are also grouped into factors strongly related to INFOR_REL, FOR_REL, CREA, IP, and SC, consistent with the theoretical predictions proposed in this study.

### 5.4. Structural Equation Modeling

The normality of the factors referring to the dependent variables was verified. The assumptions the residues of the relationships must fulfil are therefore presumed. The study of normality was carried out using the Kolmogorov–Smirnov test, with satisfactory results. All correlation coefficients are below 0.8, therefore, multicollinearity is not likely to be a concern [84].

The two types of relationships (informal and formal) have a significant and positive effect on creativity. Hypotheses H1 and H2 are confirmed. In addition, creativity explains the results of IP, but no evidence was found for R&D. Hypothesis H3, therefore, is partially confirmed. H3a is supported but H3b is not supported. 

The results show that informal relationships are relevant to the direct development of R&D, but no evidence was found for IP. Hypothesis H4 is therefore partially confirmed, and informal relationships lead the firm to excellent R&D (H4a is not supported and H4b is supported). Informal relationships are the key ingredients of long-term R&D. On the other hand, the results confirm the impact formal relationships have on IP, but not on R&D. This means that hypothesis H5 is also partially confirmed (H5a is supported but H5b is not supported).

The indices reveal a suitable overall model fit: 

1. REL-CREA-IP: The Chi-square (χ2) is 1022.486 (degrees of freedom = 270, *p* = 0.000), χ2/df has a value of 3.787 and is not much higher than 3.0 [85]. The CFI is 0.838 and the Tucker–Lewis Index (TLI) is 0.821. These scores are close to 0.9, which indicates a good fit. The RMSEA is 0.071, less than 0.08, and therefore indicates a good fit [86]. 

2. REL-CREA-R&D: The Chi-square (χ2) is 1023.651 (degrees of freedom = 270, *p* = 0.000), χ2/df has a value of 3.791. The CFI is 0.857 and the TLI is 0.841. The RMSEA is 0.077. The results present a good joint model fit.

The SEM results are presented in Table 4.

### 5.5. Structural Equation Modeling with Social Capability as Moderator

Employees that show communication, empathy, and socialize can therefore drive this behavior towards innovation success. A multigroup moderation analysis was performed in SEM, with a distinction being made between collaborators with high SC (N = 166) and those with low SC (N = 79). SC enhances and strengthens most of the effects between relationships, creativity, IP and R&D. High SC enhances the effects between informal relationships and creativity, formal relationships and creativity, creativity, and IP and formal relationships and IP. Hypothesis H6 is therefore largely fulfilled, so it is partially confirmed (Table 4).

The indices with SC have an adequate overall model fit. 

1. REL-CREA-IP (SC as moderator): The χ2 is 1338.548 (degrees of freedom = 540, *p* = 0.000), χ2/df has a value of 2.479. The CFI is 0.880 and the TLI is 0.856. These scores are close to 0.9, which indicates a good fit. The RMSEA is 0.078, less than 0.08, and therefore indicates a good fit.

2. REL-CREA-R&D (SC as moderator): The χ2 is 1326.949 (degrees of freedom = 540, *p* = 0.000), χ2/df has a value of 2.457. The CFI is 0.814 and the TLI is 0.893. The RMSEA is 0.077. The results present a good joint model fit with high SC.

**Figure 1:**
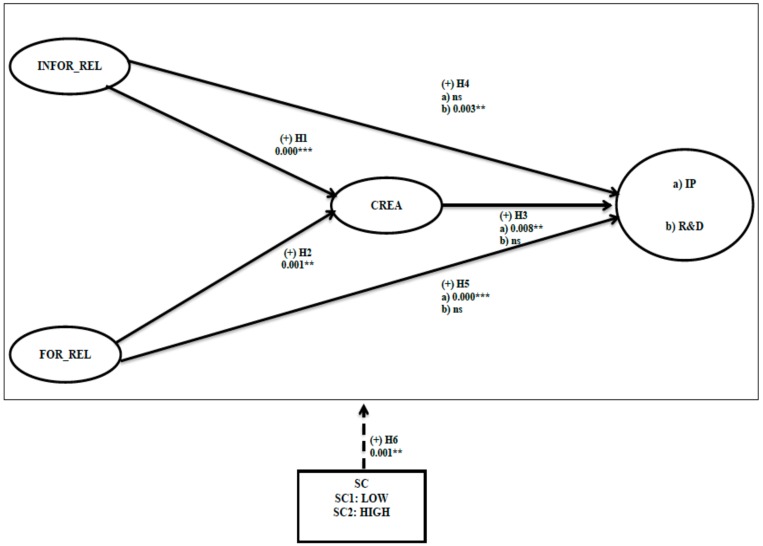
Internal social networks model predicting innovation performance and research and development.

The SEM results are presented in Figure 1.

## 6. Conclusions

This study includes an additional type of relationship, namely informal. Interesting conclusions are thus obtained about the effects each has on creativity, IP and R&D. Both types of relationships have important effects on different phases of innovation. Employees need relaxed environments and informal relationships as a source of ideas and new results. Feedback between members of firms is the most important source of creativity and innovation. Formal relationships can help develop new contracts and close new agreements. Therefore, informal relationships can be more long-term relationships because they involve a high level of trust, commitment, identification with the group, and well-being [56].

Informal relationships can help the firm to obtain excellent results within a competitive and dynamic environment, being the major driver for obtaining new customers, partners, and suppliers and, therefore, new contracts, new projects, and new products and processes. Relationships are relevant for achieving innovative solutions. A firm with a suitable system of employee relationships will be more creative, more innovative, and will be able to obtain new R&D projects.

In addition, creativity is included as an antecedent of IP and R&D, so when informal and formal relationships are antecedents of creativity, new ideas will usually generate new products, processes, or projects.

Informal relationships are a very important resource for improving IP and R&D. These relationships are the true forces for getting goals for the long term. Relationships based on trust, teamwork, friendliness, satisfaction and new approaches will therefore be a key resource for the success of a firm’s IP and R&D.

Authors such as Van de Ven [54] and Yun and Lee [72] report that when employee social skills level are high, they have more variety of tasks, they are proactive, and can generate a greater number of ideas. Therefore, it is not enough to have good formal relationships. Firms need employees with more informal relationships to be leaders in IP and R&D. The results show that the direct effect of formal relationships on IP is powerful but formal relationships have no effect on R&D. However, informal relationships have a positive and direct impact on R&D but there is no impact on IP. Both types of relationships are antecedents of creativity. The results also show that the effect of informal relationships on creativity is more powerful. This shows that creativity is a complex concept, which arises from within employees and can be transformed into a multitude of results. Furthermore, creativity can vary depending on the internal circumstances of the employees and it can also vary depending on the organizational levels (individual, group or organizational level), measures, or assessment tools [87]. 

Additionally, the model shows that the effects between relationships, creativity, IP and R&D are more solid when employees have high SC, as it helps to generate new relationships. 

Finally, our results show that having relationships between employees strengthens the development of new ideas that can generate new projects and products. Relationships, SC, and teamwork are three cornerstones for the development of large R&D projects at business, national, and international level. Due to relationships and SC, experts are collaborating together in the development of a vaccine against Covid-19, which is a great contribution of human relationships for the development of a great project of public health worldwide [14,15]. In addition, relationships not only allow us to advance in major technological or health projects, but also allow us to advance in agreements and pacts in environmental research, e.g., in the fight against climate change with the relationships and contributions of different involved agents [70].

### 6.1. Theoretical Contribution

Some studies have examined the effects between human resources and IP from a global and aggregated perspective. Others have studied the effect of formal relationships on IP. This study goes a step further by examining ambidextrous relationships as a source of creativity, IP and R&D. There are a few studies that measure and analyze informal relationships, maybe because it is very difficult to obtain information about this topic. One reason may be the high cost of obtaining information related to employees’ innermost aspects. Researchers should pay more theoretical and empirical attention to this construct from an ambidextrous perspective. It is one of the true architects of innovation within the company. As the results show, informal relationships are the most important driver of creativity and R&D, and managers should pay more theoretical and empirical attention to this construct. Collaborators with both types of relationships will lead to a greater development of novel ideas and, consequently, innovative results. The model includes employees’ most dynamic environments to support and strengthen the effects initially raised between relationships, creativity, PIP, and R&D. 

In addition, this study provides new evidence on the importance of multigroup analysis in employee relationships and innovation studies. The model analyzes a contingent effect through a multigroup analysis in SEM. 

This study therefore contributes to the literature based on Social Systems and Ambidextrous Organization. If a firm has obtained new relationships, it is easier to generate new ideas, IP, R&D, and employees’ dynamic environments strengthen these relationships. The analysis of individual creativity is very important, but few studies have examined it. Accordingly, this study investigates employee creativity as the most important antecedent of IP and R&D for two reasons: (1) Individual creativity is the first variable featuring the effects of relationships; and (2) Individual creativity is the most important antecedent of IP and R&D.

### 6.2. Managerial Implications

There are a lot of relational practices available to firm managers (e.g., meetings, networking, lunch). It is, therefore, necessary to examine different types of relationships to promote innovative activity. Managers need to know how to improve employee relationships. This study shows the importance of the most intangible part of relationships, such as informal relationships and SC, which arise from internal experience. It is the true architect of long-term value creation.

Yet, how can managers improve informal relationships? Everyone knows how to increase formal relationships (wages, teamwork, new customers, etc.), but it is not easy to have employee feedback. Maybe SC can be a cornerstone for achieving that purpose. The results show that SC is an extraordinary capability.

Managers should not consider the efforts made to stimulate creativity or SC as a cost, but instead as a long-term investment. This investment must be made continuously to become pioneers in IP and R&D. Managers must pay special attention to their collaborators’ SC. This capability helps to improve new contacts and relationships. Firms with collaborators with high SC find it easier to drive the power of relationships towards IP and R&D success.

We must point out that the firms in our study belong to 14 different industrial sectors [20]. All firms are highly innovative because they have received funding from the Spanish government during the last five years for the development of R&D projects but, depending on the industrial sector, it is easy or difficult to manage the relationships to achieve the innovation. For example, a firm located within the information and communications sector is likely to have more physical support that helps the manager to manage relationships than a firm within the agricultural sector. Although we start from the basis that all the firms in the study are highly innovative and they have sufficient potential to be able to manage properly the relationships between the employees for the development of R&D and innovation.

Finally, we provide four relevant practical recommendations and implementation guidelines for SMEs:

1. Ambidextrous relationships for R&D and sustainable innovation:
  - The willingness of managers to support new exploration and exploitation activities.
  - Attendance of congresses, fairs and meetings to obtain new trust relationships between employees.
  - Joint innovation projects with employees oriented toward exploration and exploitation activities.
2. Investment and training in new high-performance human resource practices for sustainable innovation [88]:
  - Courses and training.
  - Engaging employees’ trust and motivation for developing new relationships and ideas.
  - Creation of a culture that encourages creativity and sustainable innovation.
  - The introduction of norms, behaviors, and times that encourage creativity and sustainable innovation.
3. High social capability for sustainable innovation:
  - Climate for networking and informal meetings.
  - Encouragement for taking risks in new product development.
  - Teamwork and effective communication.
  - Involvement and help between employees.
4. New ideas for R&D and sustainable innovation:
  - Reinforcement of information sharing.
  - Creation of a culture that encourages informal relationships and formal relationships.
  - Encouragement for employees to share their knowledge and ideas.
  - Support for networking among employees (online/offline and inside/outside the company) [89].

In short, this study invites the business community to identify the effects that can be derived from ambidextrous relationships, creativity, and SC. It may lead to extraordinary innovation results within the organization and contribute to the development of large R&D projects in the field of public health and environmental research.

### 6.3. Limitations and Future Research

Considering the time and cost limitations, a sample of 245 is small (response rate is 16.94%). Nevertheless, the survey is nationwide and only includes Innovative Spanish SMEs. For this reason, results can be generalized with caution. Future studies on other countries or industries are necessary to test the results.

Longitudinal studies that incorporate several levels of analysis could provide evidence on the relationships and interactions. This study is based on three years where firms have R&D financial projects, so it would be interesting to test its important findings over a longer time. The sample focuses on firms across 14 industries with CDTI funding. Future studies could focus on examining sectorial and international effects, since firms from certain industries and countries are exposed to more opportunities for innovation.

In addition, it is necessary to mention some limitations that our models may have because they have been tested with SEM. First, in defining the model we have tried not to exclude important variables from a theoretical point of view. Our model makes a great effort to measure all the relevant variables, but as can be seen below it is always possible that some measure is being neglected. Second, our models get a good fit that does not exclude that there may be other models that can also fit the data well. It would be interesting if future research could contrast other models that may also be supported by our theoretical arguments (or by rival theoretical arguments).

We recommend future research in further examining the links between formal and informal relationships and creativity. This extension could be achieved in multiple ways. One way is to improve the measurement of creativity through a different framework and other techniques as Consensual Assessment Technique (CAT) between expert and nonexpert [90] or the new heuristic framework in three dimension of creativity (level, facet, and measures) [91]. In addition, the other variable measures may also have certain limitations if we take into account other scales or different approaches, e.g., another type of broader formal and institutional relationship (employees–company–states), another type of innovation (radical and incremental), or the SC from the point of view of international relationships, among others. Furthermore, if R&D can be included within a pre-innovation phase, future studies can test our model using a single measure for innovation. However, the study of the separate effects on each innovation type can also be very adequate. In this way, companies can focus their efforts on relationships and types of innovation where they can obtain a greater competitive advantage in the market.

Another extension would be to inspect more thoroughly informal relationships regarding creativity. It might therefore be relevant to study the effect of formal relationships from a different angle—for example, influence of leadership on employees—and not only from the point of view of employee to employee [92]. The formal and informal relationships and creativity of employees could also be studied for various periods of time.

The results suggest that an organization should be concerned about informal relationships too. In this context, we raise certain questions for future research: what happens when employees only have reached a sufficient level of formal relationships? What will the effect be? What happens when employees have reached a sufficient level of informal relationships? What will the effect be? Perhaps, once employees have enough formal relationships, organizations should invest mainly in informal relationships.

## Figures and Tables

**Table 1:** Demographic information.

Population	Highly Innovative Companies (CDTI)
Population Size	1446
Industry Categories	Agriculture and livestock (N = 4), manufacturing (N = 110), power and gas supply (N = 3), water supply and pollution (N = 3), building (N = 10), vehicle trade and repair (N = 27), transport and storage (N = 2), catering (N = 2), information and communication (N = 27), housing (N = 1), scientific activities (N = 47), administrative activities (N = 5), health activities (N = 3) and other services (N = 1)
Geographical Area and Period	Spain – 2016, 2017, and 2018
Sample Unit	Company
Information Collection	Online survey (SurveyMonkey)
Surveyed	CEO
Sample Size	245
Response Rate	16.94%
Sample Error	±5.71%
Information Sources	Primary sources: online survey and own database Secondary sources: CDTI y SABI
Statistical Software	Statistical Package for the Social Science (SPSS) and AMOS.
Empirical Analysis	Descriptive statistics, ANOVA, factor analysis, and structural equation model

**Table 2:** Loading factors.

	Loading Factor
**Relationships (V.E. = 62.22%)**	
**1.Factor: Informal Relationships (INFOR_REL)** [79] (*α* = 0.91)	
**INFOR_REL2.** Meetings	0.66
**INFOR_REL6.** Teamwork	0.50
**INFOR_REL10.** Informal meetings	0.77
**INFOR_REL11.** Discussion	0.83
**INFOR_REL12.** Agreement	0.82
**INFOR_REL13.** Shared spaces	0.59
**INFOR_REL14.** Accord	0.77
**INFOR_REL15.** Cooperation	0.83
**2.Factor: Formal Relationships (FOR_REL)** [55] (*α* = 0.88)	
**FOR_REL1.** Software and databases	0.50
**FOR_REL3.** Customers as sources of information	0.70
**FOR_REL4.** Supplier as sources of information	0.87
**FOR_REL5.** Allies as sources of information	0.74
**FOR_REL7.** Work with customers	0.72
**FOR_REL8.** Work with supplier	0.83
**FOR_REL9.** Work with allies	0.63
**Creativity (TTCT, [22]** **)**	
**(V.E=75.09%);** (*α* = 0.94)	
**CREA1.** Curiosity and pro-activity	0.90
**CREA2.** Ideas	0.92
**CREA3.** Various solutions	0.88
**CREA4.** Infrequent solutions	0.88
**CREA5.** Care, detail, and production	0.79
**CREA6.** Spontaneity and improvisation	0.85
**CREA7.** Energy and vitality	0.84
**Sustainable Product Innovation Performance** [35,79] **(V.E=73.69%);** (*α* = 0.68)	
**PIP1.** Number of innovations in product for a long time	0.78
**PIP2.** Sales of new product for a long time	0.82
**PIP3.** New Products comparison with portfolio products	0.75
**Sustainable Process Innovation Performance** [19,80] **(V.E=78.26%);** (*α* = 0.85)	
**PIP4.** Number of process innovation for a long time	0.78
**PIP5.** New processes mean less time and more productive flexibility	0.93
**PIP6.** New processes mean a reduction in costs	0.93
**Research & Development [55]**	
**(V.E=90.85%);** (*α* = 0.95)	
**R&D1.** Employees dedicated to R&D	0.96
**R&D2.** R&D expenses	0.95
**R&D3.** Hours dedicated to R & D	0.95
**Social Capability** [36] **(V.E=68.23%);** (*α* = 0.90)	
**SC_1.** Kindness	0.70
**SC_2.** Hugs	0.70
**SC_3.** Games	0.69
**SC_4.** Social norms	0.75
**SC_5.** Help	0.78
**SC_6.** Speak	0.86
**SC_7.** Verbal ability	0.80
**SC_8.** Speak first person	0.69
**SC_9.** Integration	0.85
**SC_10.** Networking	0.70

*Note:* V.E = Variance explained; *α* = Cronbach’s alpha; N = 245.

**Table 3:** Descriptive statistics and Pearson correlations matrix.

Variable	M	SD	1	2	3	4	5	6	7
Informal Relationships	5.71	1.24	1						
Formal Relationships	5.32	1.51	0.30 **	1					
Employee Creativity	5.36	1.15	0.26 **	0.47 **	1				
Product Innovation Performance	5.40	1.57	0.26 **	0.26 **	0.37 **	1			
Process Innovation Performance	5.17	1.75	0.39 **	0.34 **	0.39 **	0.33 **	1		
Research and Development	4.79	1.85	0.19 **	0.38 **	0.32 **	0.26 **	0.29 **	1	
Social Capability	5.36	1.22	0.20 **	0.45 **	0.41 **	0.32 **	0.34 **	0.46 **	1

*Note:* ** = *p* < 0.05; N = 245.

**Table 4:** Structural model fit, research hypotheses, and results.

Model 1: REL-CREA-IP/R&D	Paths	Estimate	SE	CR	*p*-Value	Results
H1 (+)	CREA ← INFOR_REL	1.254	0.145	8.649	0.000 ***	Supported
H2 (+)	CREA← FOR_REL	0.134	0.042	3.187	0.001 **	Supported
H3a (+)	IP ← CREA	0.392	0.148	2.639	0.008 **	Supported
H3b (+)	R&D ←CREA	0.098	0.195	0.501	0.617	Not Supported
H4a (+)	IP ←INFOR_REL	0.134	0.222	0.604	0.546	Not Supported
H4b (+)	R&D ← INFOR_REL	0.942	0.314	3.005	0.003 **	Supported
H5a (+)	IP ← FOR_REL	0.308	0.080	3.823	0.000 ***	Supported
H5b (+)	R&D ← FOR_REL	0.139	0.100	1.392	0.164	Not Supported
**Model 2: REL-CREA-IP/R&D (SC1 = LOW)**						
	CREA ← INFOR_REL	1.058	0.258	4.093	0.000 ***	Supported
	CREA← FOR_REL	0.113	0.076	1.489	0.136	Not Supported
	IP ← CREA	0.018	0.055	0.323	0.746	Not Supported
H6 (+)	R&D ← CREA	0.084	0.262	0.319	0.750	Not Supported
	IP ← INFOR_REL	0.021	0.081	0.256	0.798	Not Supported
	R&D ← INFOR_REL	0.773	0.424	1.826	0.068 *	Supported
	IP ← FOR_REL	0.100	0.089	1.122	0.262	Not Supported
	R&D ← FOR_REL	0.019	0.144	0.133	0.894	Not Supported
**Model 3: REL-CREA-IP/R&D (SC2 = HIGH)**						
	CREA ← INFOR_REL	1.822	0.460	3.960	0.000 ***	Supported
	CREA← FOR_REL	0.155	0.068	2.272	0.023 **	Supported
	IP ← CREA	0.514	0.202	2.540	0.011 **	Supported
H6 (+)	R&D ← CREA	0.212	0.252	0.843	0.399	Not Supported
	IP ← INFOR_REL	0.319	0.493	0.647	0.518	Not Supported
	R&D ← INFOR_REL	0.740	0.653	1.134	0.257	Not Supported
	IP ← FOR_REL	0.331	0.138	2.394	0.017 **	Supported
	R&D ← FOR_REL	0.152	0.169	0.899	0.369	Not Supported

*Note:* SE = Standard Error; CR = Composite Reliability; *** = *p* < 0.001; ** = *p* < 0.05; * = *p* < 0.1; SC1 = Low Social Capability; SC2 = High Social Capability; N = 245.
